# Transcriptional (ChIP-Chip) Analysis of ELF1, ETS2, RUNX1 and STAT5 in Human Abdominal Aortic Aneurysm

**DOI:** 10.3390/ijms160511229

**Published:** 2015-05-18

**Authors:** Matthew C. Pahl, Robert Erdman, Helena Kuivaniemi, John H. Lillvis, James R. Elmore, Gerard Tromp

**Affiliations:** 1 Sigfried and Janet Weis Center for Research, Geisinger Health System, Danville, PA 17822, USA; E-Mails: mcp3bp@virginia.edu (M.C.P.); rerdman@geisinger.edu (R.E.); shkuivaniemi@geisinger.edu (H.K.); 2 Department of Surgery, Temple University School of Medicine, Philadelphia, PA 19140, USA; 3 Department of Ophthalmology, Wayne State University School of Medicine, Detroit, MI 48202, USA; E-Mail: johnlillvis@gmail.com; 4 Department of Vascular and Endovascular Surgery, Geisinger Health System, Danville, PA 17822, USA; E-Mail: jelmore@geisinger.edu

**Keywords:** aneurysm, aorta, genes, transcription factor, chromatin immunoprecipitation, gene expression, gene ontology, KEGG pathway, network

## Abstract

We investigated transcriptional control of gene expression in human abdominal aortic aneurysm (AAA). We previously identified 3274 differentially expressed genes in human AAA tissue compared to non-aneurysmal controls. Four expressed transcription factors (ELF1, ETS2, STAT5 and RUNX1) were selected for genome-wide chromatin immunoprecipitation. Transcription factor binding was enriched in 4760 distinct genes (FDR < 0.05), of which 713 were differentially expressed in AAA. Functional classification using Gene Ontology (GO), KEGG, and Network Analysis revealed enrichment in several biological processes including “leukocyte migration” (FDR = 3.09 × 10^−05^) and “intracellular protein kinase cascade” (FDR = 6.48 × 10^−05^). In the control aorta, the most significant GO categories differed from those in the AAA samples and included “cytoskeleton organization” (FDR = 1.24 × 10^−06^) and “small GTPase mediated signal transduction” (FDR = 1.24 × 10^−06^). Genes up-regulated in AAA tissue showed a highly significant enrichment for GO categories “leukocyte migration” (FDR = 1.62 × 10^−11^), “activation of immune response” (FDR = 8.44 × 10^−11^), “T cell activation” (FDR = 4.14 × 10^−10^) and “regulation of lymphocyte activation” (FDR = 2.45 × 10^−09^), whereas the down-regulated genes were enriched in GO categories “cytoskeleton organization” (FDR = 7.84 × 10^−05^), “muscle cell development” (FDR = 1.00 × 10^−04^), and “organ morphogenesis” (FDR = 3.00 × 10^−04^). Quantitative PCR assays confirmed a sub-set of the transcription factor binding sites including those in *MTMR11*, *DUSP10*, *ITGAM*, *MARCH1*, *HDAC8*, *MMP14*, *MAGI1*, *THBD* and *SPOCK1*.

## 1. Introduction

Abdominal aortic aneurysms (AAAs) are a chronic disease whose pathogenesis is poorly understood [1,2,3,4,5,6]. The lack of knowledge about the underlying molecular mechanisms is hampering the development of treatment modalities. Currently surgical intervention is successful, but is used at a late stage of the disease leaving patients with small AAAs in the situation of “watchful waiting” until the AAA has grown large enough to require surgery. Slowing down growth of AAA will make many operations unnecessary. Development of methods to slow down the growth of small AAAs requires better understanding of the molecular pathways involved in this process.

We previously carried out a whole-genome microarray-based expression analysis comparing aortic tissue samples obtained from patients with AAAs to those without the disease and identified 3274 genes whose expression was significantly decreased (*n* = 1793) or increased (*n* = 1481) in the AAA tissue [7]. *In silico* analysis of the promoters of the up-regulated genes showed that they were enriched for binding sites of a small number (*n* = 13) of transcription factors (TFs), suggesting co-regulation and a significant role in the pathogenesis of AAAs [8]. Protein expression of the TFs binding to these sites was confirmed in control aorta and AAA tissue by immunohistochemical staining [8].

Understanding the transcriptional networks that control human AAA development requires experimental cataloging of the target genes of each TF [9,10,11]. In the current study we carried out further analyses with four of the previously identified TFs (ELF1, ETS2, RUNX1 and STAT5; Table 1) predicted to control the expression of differentially expressed genes in AAA. Two of the TFs studied here, ELF1 and ETS2, are members of the highly evolutionarily conserved Ets family of TFs that contains an 85 amino acids long motif called the ETS domain [12]. Unlike other TFs, the Ets family tends to form complexes with members of other TF families rather than with one another [13]. The binding behavior of ELF1 and ETS2 differs in that ELF1 binds strictly to its core motif, while ETS2 binds weakly to its targets [13]. Both ELF1 and ETS2 are expressed in human AAA and non-aneurysmal infrarenal aorta with ELF1 showing significantly increased expression in AAA (Table 1).

**Table 1:** mRNA and protein expression of transcription factors ELF1, ETS2, RUNX1 and STAT5 in human aneurysmal and non-aneurysmal infrarenal aorta.

TF	Gene ID	mRNA Levels *		Protein Expression *	
		AAA *vs.* Control	FDR	AAA	Control
**ELF1**	1997	1.8-fold increase	0.0263	+++	++
**ETS2**	2114	1.4-fold decrease	NS	++	++
**RUNX1**	861	2.5-fold increase	0.0058	++	+
**STAT5**	6776 ( *STAT5A*)	No difference	NS	+++	++
	6777 ( *STAT5B*)				

* These results are based on our previous studies in which mRNA levels were assessed by microarray analysis [7] and protein levels by immunohistochemical analyses [8,14]; NS: Not significant; +: Detectable, low level of expression; ++: Moderate level of expression; +++: High level of expression.

The third TF found to have a role in the transcriptional control of gene expression in AAA is AML1, acute myeloid leukemia 1 (NCBI official symbol: RUNX1, runt-related transcription factor 1, also called EVI-1), which belongs to a complex of two TFs (Moloney murine leukemia virus enhancer core binding factor (CBF) and polyomavirus enhancer binding protein 2; PEBP2) [15]. Its mRNA levels were significantly increased in AAA tissue (Table 1). RUNX1, which plays a role in hematopoietic development through specifying the hematopoietic stem cell [16], has been studied extensively in leukemia due to chromosome 21 to chromosome 8 translocation that is often found in leukemia, but is by itself insufficient to cause leukemia in mouse models [17]. Knock out of *Runx1* in early stages of development results in an embryologically lethal phenotype in mice; knock out in later stages results in abnormalities in hematopoiesis [18]. RUNX1 has three protein coding isoforms (RUNX1A, RUNX1B, and RUNX1C), each of which has different roles in embryonic hematopoiesis [16]. Post-translational modification of RUNX1 affects its affinity to its targets [19].

The fourth TF studied here, STAT5 (signal transducer and activator of transcription 5) was found to play a significant role in the transcriptional regulation of many members of the complement cascade [14]. It is stimulated in the JAK-STAT pathway, where a trans-membrane binding protein will bind to a cytokine (interleukins, erythropoietin, thrombopoietin, growth hormone, and prolactin [20]) which results in a conformational change of the receptor’s intercellular region that induces the phosphorylation of the STAT protein by Janus kinase (JAK), which can lead to the formation of a dimer (STAT5A/B) or a tetramer (STAT5A).

The current study presents the results from analyses using chromatin immunoprecipitation followed by microarray hybridization (ChIP-chip). Independent experiments were carried out with the four TFs—ELF1, ETS2, RUNX1 and STAT5—in human AAA and non-aneurysmal aortic tissue. Combined analysis with ChIP-chip and genome-wide mRNA expression data on human AAA tissue was also carried out to provide biologically relevant and disease-centered information.

## 2. Results and Discussion

### 2.1. Expression of TFs by Cultured Aortic and Inflammatory Cells

Before experimenting with hard-to-get human aortic tissue samples, we carried out experiments with human cell lines representing the different types of cells present in the aortic wall. We used commercially available human aortic smooth muscle (HASMC), endothelial (HAEC) and macrophage (THP-1) cell lines. In disease states, such as AAA, cells in the aortic wall are exposed to different stressors including cytokines, reactive oxygen species, and matrix metalloproteinases. To mimic this environment, we cultured the cells in the presence or absence of lipopolysaccharides (LPS) or interferon γ (IFNγ), which have been shown to activate these cells [21,22].

**Figure 1:**
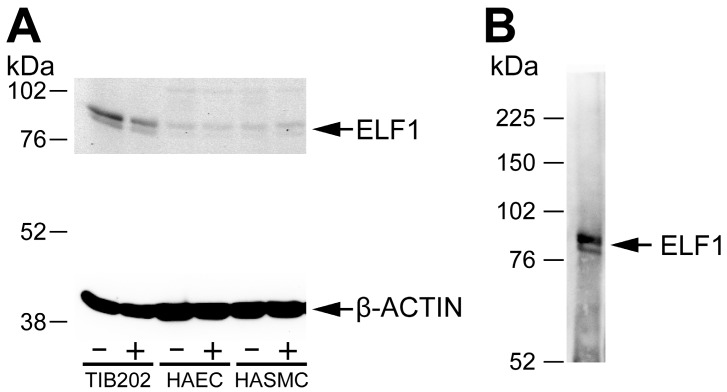
Western blots of (**A**) protein lysates from three different cell lines and (**B**) immunoprecipitated proteins from a macrophage cell line THP-1. (**A**) Three different human cell types (macrophage, THP-1 also called TIB202; aortic endothelial, HAEC; and aortic smooth muscle, HASMC), were grown either unstimulated (−) or stimulated (+) with LPS or IFNγ, harvested, lysed and analyzed in Western blot using a commercially available ChIP-validated antibody against ELF1 [23]. The lower part of the blot was incubated with β-actin antibody to use as a loading control. The β-actin antibody produced a strong band of the expected size (42 kDa); (**B**) THP-1 cells were immunoprecipitated using the ELF1 antibody and the precipitated proteins detected in a Western blot against the same ELF1 antibody. The sizes of molecular weight markers are shown in kDa. The expected sizes of the cytoplasmic and nuclear form of ELF1 are 80 and 98 kDa, respectively.

We tested antibody specificity to ensure that we can identify binding sites specific for ELF1, ETS2, and RUNX1, the TFs chosen for the study based on our previous *in silico* analyses [8], as well as STAT5 identified as a key regular in our previous study on the complement cascade [14]. Figure 1 shows results using an antibody against ELF1 [23,24] in a Western blot against protein lysates obtained from the cell cultures (A), as well as an immunoprecipitation with this antibody, followed by Western using the same antibody (B). The immunoprecipitation was carried out using the same protocol as in ChIP except not doing the crosslinking step. The results demonstrated the specificity of the ELF1 antibody, and verified the expression of ELF1 protein by the cell lines. The stimulation of these cell lines with LPS or IFNγ led to an increase in the amount of ELF1 protein (1.8-fold increase in the nuclear form in HASMC when standardized with β-actin protein levels). Similar experiments were carried out with antibodies against ETS2 [24,25,26], RUNX1 [27] and STAT5 [14] (not shown). All four TFs were expressed in all the cell lines (not shown).

### 2.2. Analysis of ChIP-Chip Results from Aortic Tissue

The ChIP-chip for aortic samples (Table 2) was carried out in two pools of samples, “the AAA Pool” (combining six AAA tissue samples) and “the Control Pool” (combining five control tissue samples), due to the large amount of tissue required for the experiment. Each TF was analyzed in a separate experiment. The ChIP-enriched regions (chers), in the human genome are listed in Table S1. After identifying the chers, we identified the genes in whose promoter regions (10 kb upstream of the transcription start site (TSS), or within the gene) the chers were located. The number of genes with chers per TF was similar in AAA (1330 to 1513) and control (1491 to 1570) tissues, and between the different ChIP experiments carried out with antibodies against different TFs (Table 3). There was, however, only a small overlap of 147 to 175 genes between the AAA and control samples in different experiments (Table 3). We examined the sequences of the chers to identify TF binding motifs, and found that approximately 76.4% of the chers contained transcription factor binding sites (TFBSs). All identified TFBSs are listed in Table S2.

**Table 2:** Donor information for aortic tissue samples.

Case ID	Age (Years)	Sex	Diagnostic Group	Cause of Death *
GHS01	67	Male	AAA	NA
GHS03	88	Male	AAA	NA
GHS07	65	Female	AAA	NA
GHS13	78	Male	AAA	NA
GHS14	75	Male	AAA	NA
GHS15	66	Male	AAA	NA
ME-02-04	50	Male	Control	Drug Overdose
ME-02-05	78	Male	Control	Cardiovascular
ME-05-01	69	Female	Control	Trauma
ME-05-03	54	Male	Control	Cardiovascular
ME-10-02	53	Male	Control	Cardiovascular

* Cause of death is given for controls; NA, not applicable, since aortic tissue samples from AAA patients were obtained during surgical repairs. Summary statistics for the AAA and control groups: AAA group (*n* = 6): mean age = 73.2 ± 9.0; median = 71; male:female = 5:1; and control group (*n* = 5): mean age = 60.8 ± 12.1; median = 54; male:female = 4:1.

After identifying the chers with motifs for the TFBSs, we determined the distance from the predicted site to the TSS (Table S2). We used the most proximal TSS for each gene, and found 4760 known genes that were enriched in AAA, 713 of which were differentially expressed in the previous mRNA expression study (Table S3) [7]. In addition we sampled control aortic tissue and found chers associated with 4985 known genes with 680 genes differentially expressed [7].

The ChIP-chip data for the stimulated cultured mononuclear cells were analyzed using same methods as the tissue data. Overall we found fewer genes expressed in the mononuclear blood cell cultures compared to the tissue samples. Since none of the genes were enriched in both the cell and tissue experiments (not shown), further analyses were carried out with only the aortic tissue samples.

**Table 3:** Number of genes with chers, genes with differential expression in AAA, and chers with TFBSs.

Transcription Factor	Genes with Chers *		Genes with Chers in both AAA and Control		Differentially Expressed Genes †			Chers with TFBS N (%)
	AAA N	Control N	N	%	AAA N	Control N	Overlap N (%)	
RUNX1	1330	1491	147	5.2	209	186	18 (4.6)	80.0
ELF1	1513	1570	166	5.4	217	216	28 (6.5)	81.3
ETS2	1448	1539	175	5.9	201	215	30 (7.2)	79.0
STAT5	1490	1557	151	5.0	221	209	23 (5.3)	79.1

* ChIP-enriched regions (cher) were identified as regions containing at least seven probes with a mean enrichment value that exceeded the threshold (*y*_0_) and that were separated by at least 450 bp from another cher. The threshold was defined as the top 99th quantile (top 1%) of potential chers to limit the number of false positives included in the analysis; **^†^** The genome-wide microarray-based mRNA expression data for aneurysmal and non-aneurysmal human infrarenal abdominal aorta can be obtained from the Gene Expression Omnibus (GEO) database (Series# GSE7084) [28,29], and were described in detail in a previous publication [7]. For a listing of chers and TFBS, see Tables S1 and S2, respectively. Abbreviations: AAA: abdominal aortic aneurysm; TFBS: transcription factor binding site.

### 2.3. Validation of ChIP-Chip Results by qPCR

Ten genomic regions containing TFBSs predicted from ChIP-chip results and in target genes (*MTMR11*, *DUSP10* (two regions), *ITGAM*, *MARCH1*, *HDAC8*, *MMP14*, *MAGI1*, *THBD* and *SPOCK1*) were validated using specific qPCR assays (Table 4). For all of the 10 regions, ChIP from the AAA sample showed more binding than the control aorta in both ChIP-chip and qPCR. Based on the consensus binding site sequences, the regions in *MTMR11* (binding site for RUNX1), *MAGI1* (binding site for STAT5), *THBD* (binding site for ETS2) and *SPOCK1* (binding site for ETS2) contain multiple potential sites within the approximately 1 kbp qPCR product.

**Table 4:** List of qPCR assays used in the study.

TF	Gene	Chr.	ChIP-Chip Results					qPCR Assay			
			CHER Start	CHER End	Max Peak	Score		TFBS	SAB Catalog Number	Position	Delta
**RUNX1**	*MTMR11*	1	149914894	149915698	1.82	5.51		149914981	GPH1014896	149915029	48
								149914982	GPH1014896	149915029	47
								149915422	GPH1014896	149915029	−393
								149915646	GPH1014896	149915029	−617
**ELF1**	*DUSP10*	1	221910850	221912636	1.11	3.48		221911869	GPH1015368	221911302	−567
								221911869	GPH1015369	221911004	−865
**ELF1**	*ITGAM*	16	31265046	31265641	1.77	3.85		31265104	GPH1005092	31265074	−30
**ETS2**	*MARCH1*	4	164540016	164540804	1.82	7.09		164540319	GPH1023955	164540062	−257
**ETS2**	*HDAC8*	X	71795290	71796102	1.45	3.99		71795629	GPH1027352	71795212	−417
**ETS2**	*MMP14*	14	23304660	23305370	1.52	4		23305194	GPH1003823	23304254	−940
**STAT5**	*MAGI1*	3	66027042	66027848	2.14	6.17		66027092	GPH1023100	66026742	−350
								66027435	GPH1023100	66026742	−693
								66027447	GPH1023100	66026742	−705
								66027779	GPH1023100	66026742	−1037
								66027791	GPH1023100	66026742	−1049
**ETS2**	*THBD*	20	23032992	23033730	1.48	4.9		23033025	GPH1022015	23032818	−207
								23033065	GPH1022015	23032818	−247
								23033114	GPH1022015	23032818	−296
								23033328	GPH1022015	23032818	−510
**ETS2**	*SPOCK1*	5	136837910	136838620	1.2	3.02		136838118	GPH1024386	136837303	−815
								136838136	GPH1024386	136837303	−833

TF, transcription factor; Chr, chromosomal location of the gene; TFBS, location of the transcription factor binding site; CHER Start, location of the start of the ChIP-enriched region; CHER End, location of the end of the ChIP-enriched region. All qPCR assays are commercially available from SABiosciences [30] with the provided catalog numbers. Each qPCR primer pair amplifies a product of about 1 kbp. Delta, difference in bp between the predicted TFBS and the SAB assay location (middle point of the PCR product).

### 2.4. Functional Classification of TF Target Genes

**Figure 2:**
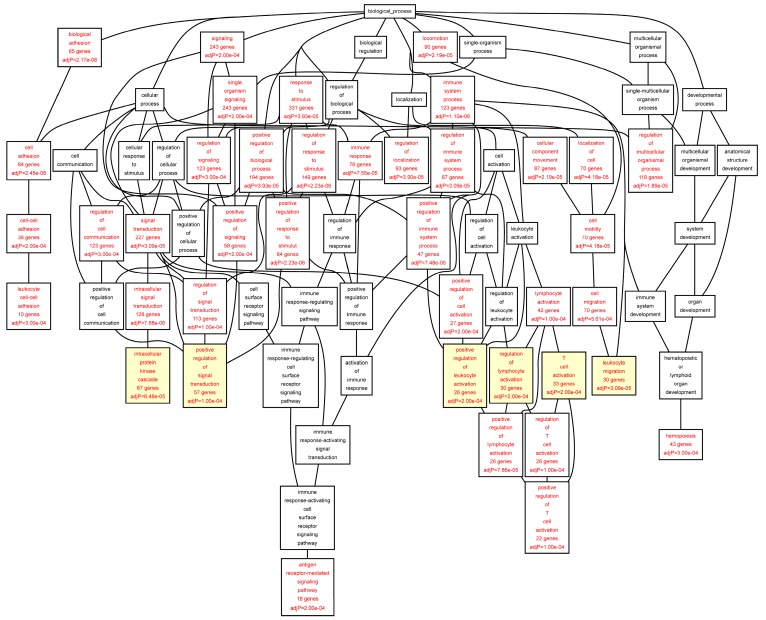
Biological categories of the differentially expressed target genes of transcription factors RUNX1, ELF1, ETS2 and STAT5 in the AAA tissue. A DAG of the GO categories of the set of 711 genes containing at least one cher in AAA tissue was generated by the web application WebGestalt [32]. Categories shown in red were significant (adjusted *p* < 0.001). The categories on the 6th level of hierarchy are discussed in the text and are shown here with a yellow background. See Table 5 and Table S4 for additional information.

**Figure 3:**
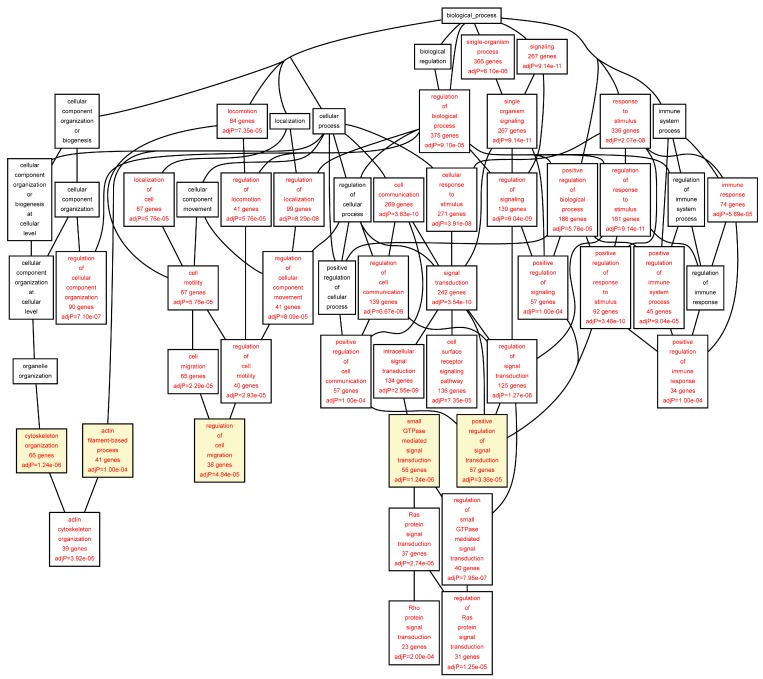
Biological categories of the differentially expressed target genes of transcription factors RUNX1, ELF1, ETS2 and STAT5 in the control aorta. A DAG of the GO categories of the set of 679 genes containing at least one cher in control tissue was generated by the web application WebGestalt [32]. Categories shown in red were significant (adjusted *p* < 0.001). The categories on the 6th level of hierarchy are discussed in the text and are shown here with a yellow background. See Table 6 and Table S5 for additional information.

**Figure 4:**
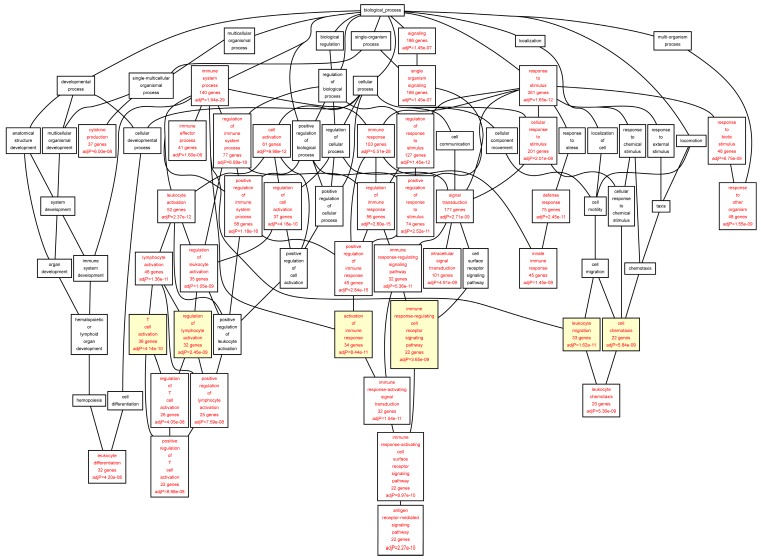
Biological categories of the up-regulated target genes of transcription factors RUNX1, ELF1, ETS2 and STAT5 in the AAA tissue. A DAG of the GO categories of the set of 395 genes was generated by the web application WebGestalt [32]. Categories shown in red were significant (adjusted *p* < 0.001). The categories on the 6th level of hierarchy are discussed in the text and are shown here with a yellow background. See Table 7 and Table S6 for additional information.

**Figure 5:**
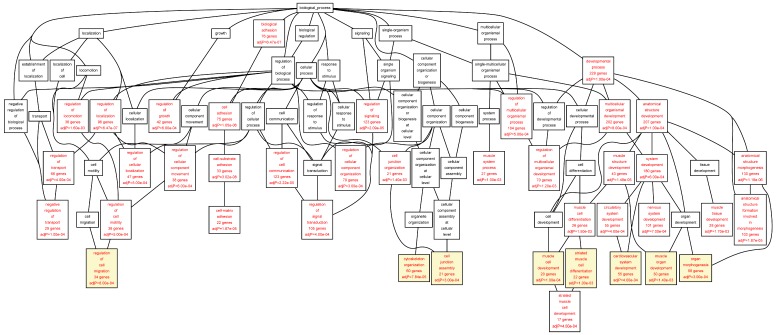
Biological categories of the down-regulated target genes of transcription factors RUNX1, ELF1, ETS2 and STAT5 in the AAA tissue. A DAG of the GO categories of the set of 551 genes was generated by the web application WebGestalt [32]. Categories shown in red were significant (adjusted *p* < 0.001). The categories on the 6th level of hierarchy are discussed in the text and are shown here with a yellow background. See Table 8 and Table S7 for additional information.

The target genes of the TFs identified in the ChIP-chip experiments were annotated using the Gene Ontology (GO) biological function categories (Table 5, Table 6, Table 7, Table 8, Table 9 and Tables S4–S8). All GO biological function categories can be found in Figure 2, Figure 3, Figure 4, Figure 5. To provide a balance between general and specific features, we discuss here the GO categories from the 6th level of hierarchy from all the analyses (Table 5, Table 6, Table 7, Table 8, Table 9). A significant enrichment of genes belonging to several GO categories was found (Table 5, Table 6, Table 7, Table 8, Table 9; Figure 2, Figure 3, Figure 4, Figure 5). In the AAA samples most significantly enriched biological function categories were “*leukocyte migration*” (FDR = 3.09 × 10^−05^), and “*intracellular protein kinase cascade*” (FDR = 6.48 × 10^−05^) (Table 5; Figure 2). In the control aorta, the most significant GO categories differed from those in the AAA samples and included “*cytoskeleton organization*” (FDR = 1.24 × 10^−06^), and “*small GTPase mediated signal transduction*” (FDR = 1.24 × 10^−06^) (Table 6; Figure 3). The GO category “*positive regulation of signal transduction*” was enriched in both AAA and control aorta (Table 5 and Table 6, Figure 2 and Figure 3).

When we analyzed the target genes separately based on their mRNA levels in the AAA tissue, genes up-regulated in AAA tissue [7] showed a highly significant enrichment for GO categories “*leukocyte migration*” (FDR = 1.62 × 10^−11^), “*activation of immune response*” (FDR = 8.44 × 10^−11^), “*T cell activation*” (FDR = 4.14 × 10^−10^), and “*regulation of lymphocyte activation*” (FDR = 2.45 × 10^−09^) (Table 7; Figure 4). The genes down-regulated in AAA were enriched in completely different GO categories including “*cytoskeleton organization*” (FDR = 7.84 × 10^−05^), “*muscle cell development*” (FDR = 1.00 × 10^−04^), “*organ morphogenesis*” (FDR = 3.00 × 10^−04^), and “*cell junction assembly*” (FDR = 3.00 × 10^−04^) (Table 8, Figure 5).

We noticed that there was substantial overlap in GO categories for the individual TFs for all but ETS2. We therefore performed a GO analysis in which the target genes of ETS2 were omitted and only the target genes for RUNX1, STAT5 and ELF1 were included. The most significantly enriched categories were “*leukocyte migration*” (FDR = 9.60 × 10^−05^), “*positive regulation of leukocyte proliferation*”, “*T cell activation*”, “*cell chemotaxis*” and “*intracellular protein kinases cascade*” (all with FDR = 8.00 × 10^−04^) (Table 9). The results were very similar to that obtained with all the TFs (Table 5), suggesting that RUNX1, STAT5 and ELF1 contribute most to the cellular immune response (T cell activation, lymphocyte and leukocyte activation and proliferation) in the AAA tissue.

**Table 5:** Enriched Gene Ontology (GO) biological process categories for the target genes identified in AAA ChIP-chip with transcription factors RUNX1, ELF1, ETS2 and STAT5.

GO Biological Process Category	GO ID	Number of Genes			*p*	FDR
		Reference Set	Aorta Set	Expected		
Leukocyte migration	0050900	248	30	3	1.21 × 10^−07^	3.09 × 10^−05^
Intracellular protein kinase cascade	0007243	867	67	34	4.17 × 10^−07^	6.48 × 10^−05^
Positive regulation of signal transduction	0009967	708	57	29	8.99 × 10^−07^	1.00 × 10^−04^
Positive regulation of leukocyte activation	0002696	230	26	9	3.04 × 10^−06^	2.00 × 10^−04^
Regulation of lymphocyte activation	0051249	287	30	12	2.75 × 10^−06^	2.00 × 10^−04^
T cell activation	0042110	332	33	2	2.68 × 10^−06^	2.00 × 10^−04^

The gene set enriched in the ChIP-chip experiments and differentially expressed based on mRNA expression profiles of the AAA [7] was compared to the reference gene set in the human genome annotated to at least one GO category. The analysis was carried out using a set of differentially expressed target genes with chers in the AAA sample pool. The analyses presented here were carried out using the combined set of all target genes for all four transcription factors. All of the categories listed here are from the 6th level of hierarchy. Size of the category (number of genes) and the number of genes overlapping with the list of genes are given. The analysis was carried out using the Webgestalt tool [31,32]. *p* values were calculated using a hypergeometric test and adjustment for multiple hypothesis testing (FDR) was performed using Benjamini-Hochberg correction [33]. See Figure 2 for a DAG image of the categories, and Table S4 for a list of the genes in the aorta set in each category.

**Table 6:** Enriched GO biological process categories for the target genes identified in control aorta ChIP-chip with transcription factors RUNX1, ELF1, ETS2 and STAT5.

GO Biological Process Category	GO ID	Number of Genes			*p*	FDR
		Reference Set	Aorta Set	Expected		
Cytoskeleton organization	0007010	792	66	31	7.57 × 10^−09^	1.24 × 10^−06^
Small GTPase mediated signal transduction	0007264	604	55	24	7.27 × 10^−09^	1.24 × 10^−06^
Positive regulation of signal transduction	0009967	708	57	28	2.77 × 10^−07^	3.36 × 10^−05^
Regulation of cell migration	0030334	395	38	16	4.33 × 10^−07^	4.84 × 10^−05^
Actin-filament-based process	0030029	463	41	18	1.34 × 10^−06^	1.00 × 10^−04^

See footnote to Table 5 for description of approach and tools used. See Figure 3 for a DAG image of the categories, and Table S5 for a list of the genes in the aorta set in each category.

**Table 7:** Enriched GO biological process categories for the up-regulated target genes identified in AAA ChIP-chip with transcription factors RUNX1, ELF1, ETS2 and STAT5.

GO Biological Process Category	GO ID	Number of Genes			*p*	FDR
		Reference Set	Aorta Set	Expected		
Leucocyte migration	0050900	248	33	7	9.09 × 10^−14^	1.62 × 10^−11^
Activation of immune response	0002253	281	34	8	6.18 × 10^−13^	8.44 × 10^−11^
T cell activation	0042110	332	36	9	3.39 × 10^−12^	4.14 × 10^−10^
Regulation of lymphocyte activation	0051249	287	32	8	2.74 × 10^−11^	2.45 × 10^−09^
Immune response-regulating cell surface receptor signaling pathway	0002768	139	22	6	4.40 × 10^−11^	3.65 × 10^−09^
Cell chemotaxis	0060326	143	22	4	7.80 × 10^−11^	5.84 × 10^−09^

See footnote to Table 5 for description of approach and tools used. See Figure 4 for a DAG image of the categories, and Table S6 for a list of the genes in the aorta set in each category.

**Table 8:** Enriched GO biological process categories for the down-regulated target genes identified in AAA ChIP-chip with transcription factors RUNX1, ELF1, ETS2 and STAT5.

GO Biological Process Category	GO ID	Number of Genes			*p*	FDR
		Reference Set	Aorta Set	Expected		
Cytoskeleton organization	0007010	792	60	30	3.31 × 10^−07^	7.84 × 10^−05^
Muscle cell development	0055001	145	20	6	6.94 × 10^−07^	1.00 × 10^−04^
Organ morphogenesis	0009887	802	58	31	2.35 × 10^−06^	3.00 × 10^−04^
Cell junction assembly	0034329	170	21	7	2.30 × 10^−06^	3.00 × 10−04
Cardiovascular system development	0072358	754	55	29	3.37 × 10^−06^	4.00 × 10^−04^
Regulation of cell migration	0030334	395	34	15	9.72 × 10^−06^	8.00 × 10^−04^
Striated muscle cell differentiation	0051146	204	22	8	1.24 × 10^−05^	1.00 × 10^−03^
Muscle organ development	0007517	340	30	13	2.03 × 10^−05^	1.40 × 10^−03^

See footnote to Table 5 for description of approach and tools used. See Figure 5 for a DAG image of the categories, and Table S7 for a list of the genes in the aorta set in each category.

**Table 9:** Enriched GO biological process categories for the target genes identified in AAA ChIP-chip with a combined analysis of transcription factorsRUNX1, STAT5 and ELF1.

GO Biological Process Category	GO ID	Number of Genes			*p*	FDR
		Reference Set	Aorta Set	Expected		
Leukocyte migration	0050900	248	26	8.3	2.44 × 10^−07^	9.60 × 10^−05^
Positive regulation of leukocyte proliferation	0070665	102	14	3.4	7.05 × 10^−06^	8.00 × 10^−04^
Cell chemotaxis	0060326	143	17	4.8	5.59 × 10^−06^	8.00 × 10^−04^
T cell activation	0042110	332	28	11	6.66 × 10^−06^	8.00 × 10^−04^
Intracellular protein kinase cascade	0007243	867	54	29	6.51 × 10^−06^	8.00 × 10^−04^
Positive regulation of signal transduction	0009967	708	46	24	1.14 × 10^−05^	1.00 × 10^−03^
Regulation of lymphocyte activation	0051249	287	25	9.6	1.20 × 10^−05^	1.00 × 10^−03^

The analysis was as described in the footnote to Table 5 except that the combined set of all target genes for the three transcription factors RUNX1, STAT5 and ELF1 were used. See Table S8 for a list of the genes in the aorta set in each category.

The differentially expressed TF target genes were also mapped to KEGG pathways (Table 10 and Table 11). In the AAA samples, the most enriched pathways were “*primary immunodeficiency*”, “*amoebiasis*”, “*hematopoietic cell lineage*”, and “*B cell receptor signaling pathway*” (Table 10), whereas in the control aorta “*Fc γ R-mediated phagocytosis*”, “*bacterial invasion of epithelial cells*”, “*viral myocarditis*” and “*arrhythmogenic right ventricular cardiomyopathy*” were the top pathways (Table 11).

**Table 10:** Enriched KEGG pathways for the target genes identified in ChIP-chip with human AAA tissue and transcription factors RUNX1, ELF1, ETS2 and STAT5.

KEGG ID	n	Expected	N	*p*-Value	Pathway Name
hsa05340	9	1.89	35	0.00001	Primary immunodeficiency
hsa05146	14	5.74	106	0.00052	Amoebiasis
hsa04640	12	4.76	88	0.00081	Hematopoietic cell lineage
hsa04662	10	4.06	75	0.0021	B cell receptor signaling pathway
hsa05414	11	4.87	90	0.0031	Dilated cardiomyopathy
hsa05410	10	4.49	83	0.0048	Hypertrophic cardiomyopathy (HCM)
hsa04510	19	10.82	200	0.005	Focal adhesion
hsa05150	7	3.03	56	0.010	Staphylococcus aureus infection
hsa05142	11	5.63	104	0.010	Chagas disease (American trypanosomiasis)
hsa04614	3	0.92	17	0.011	Renin-angiotensin system
hsa04330	6	2.54	47	0.012	Notch signaling pathway
hsa04666	10	5.14	95	0.013	Fc γ R-mediated phagocytosis
hsa05222	9	4.60	85	0.016	Small cell lung cancer
hsa04520	8	3.95	73	0.016	Adherens junction
hsa05220	8	3.95	73	0.016	Chronic myeloid leukemia
hsa00740	2	0.60	11	0.019	Riboflavin metabolism
hsa04380	12	6.93	128	0.020	Osteoclast differentiation
hsa04810	18	11.58	214	0.022	Regulation of actin cytoskeleton
hsa05214	7	3.52	65	0.023	Glioma
hsa04670	11	6.33	117	0.024	Leukocyte transendothelial migration
hsa04960	5	2.27	42	0.024	Aldosterone-regulated sodium reabsorption
hsa01040	3	1.14	21	0.024	Biosynthesis of unsaturated fatty acids
hsa04664	8	4.28	79	0.026	Fc epsilon RI signaling pathway
hsa00524	1	0.27	5	0.026	Butirosin and neomycin biosynthesis
hsa04142	11	6.55	121	0.030	Lysosome
hsa04660	10	5.85	108	0.031	T cell receptor signaling pathway
hsa04514	12	7.36	136	0.032	Cell adhesion molecules (CAMs)
hsa05221	6	3.14	58	0.036	Acute myeloid leukemia
hsa05100	7	3.84	71	0.037	Bacterial invasion of epithelial cells
hsa00061	1	0.32	6	0.038	Fatty acid biosynthesis
hsa04512	8	4.60	85	0.039	ECM-receptor interaction
hsa04722	11	6.87	127	0.041	Neurotrophin signaling pathway
hsa05140	7	3.95	73	0.042	Leishmaniasis
hsa04012	8	4.71	87	0.045	ErbB signaling pathway
hsa05412	7	4.01	74	0.045	Arrhythmogenic right ventricular cardiomyopathy (ARVC)
hsa05200	24	17.70	327	0.049	Pathways in cancer
hsa05131	6	3.36	62	0.049	Shigellosis
hsa00534	3	1.41	26	0.049	Glycosaminoglycan biosynthesis—Heparan sulfate
hsa04062	15	10.23	189	0.049	Chemokine signaling pathway

A total of 319 target genes identified in ChIP-chip with human AAA tissue had KEGG categories. n, number of genes in the experimental gene list belonging to the KEGG pathway; N, all genes in this KEGG pathway.

**Table 11:** Enriched KEGG pathways for the target genes identified in ChIP-chip with human non-aneurysmal infrarenal aorta and transcription factors RUNX1, ELF1, ETS2 and STAT5.

KEGG ID	n	Expected	N	*p*-Value	Pathway Name
**hsa04666**	13	5.29	95	0.00070	Fc γ R-mediated phagocytosis
**hsa05100**	10	3.95	71	0.00170	Bacterial invasion of epithelial cells
**hsa05416**	9	4.01	72	0.0061	Viral myocarditis
**hsa05412**	9	4.12	74	0.0074	Arrhythmogenic right ventricular cardiomyopathy (ARVC)
**hsa00640**	5	1.78	32	0.0075	Propanoate metabolism
**hsa04810**	20	11.91	214	0.0076	Regulation of actin cytoskeleton
**hsa04662**	9	4.17	75	0.0081	B cell receptor signaling pathway
**hsa05340**	5	1.95	35	0.012	Primary immunodeficiency
**hsa04510**	18	11.13	200	0.015	Focal adhesion
**hsa05410**	9	4.62	83	0.016	Hypertrophic cardiomyopathy (HCM)
**hsa04520**	8	4.06	73	0.019	Adherens junction
**hsa05414**	9	5.01	90	0.027	Dilated cardiomyopathy
**hsa00524**	1	0.28	5	0.028	Butirosin and neomycin biosynthesis
**hsa05110**	6	3.01	54	0.029	Vibrio cholerae infection
**hsa05120**	7	3.78	68	0.034	Epithelial cell signaling in Helicobacter pylori infection
**hsa00061**	1	0.33	6	0.040	Fatty acid biosynthesis

A total of 328 target genes identified in ChIP-chip with human control aorta tissue had KEGG categories. n, number of genes in the experimental gene list belonging to the KEGG pathway; N, all genes in this KEGG pathway.

**Figure 6:**
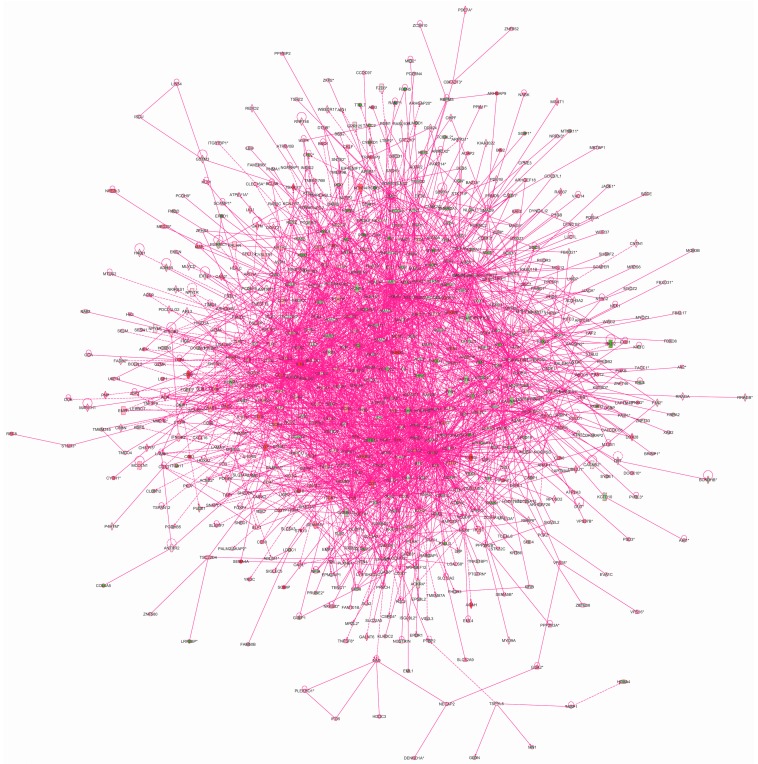
Network of interactions of the transcription factor target genes. Qiagen’s Ingenuity Pathway Analysis^®^ tool was used to analyze the genes with chers based on the current study using ChIP-chip. This analysis yielded 25 networks, which were then merged. Green molecules showed decreased and red increased mRNA expression in AAA in our previous microarray expression analysis [7]. Molecules are represented as nodes, and the biological relationship between two nodes is represented as a line. Solid lines represent direct interactions and dashed lines indirect interactions. All lines are supported by at least one literature citation or from canonical information stored in the Ingenuity Pathways Knowledge Base. Nodes are displayed using various shapes that represent the functional class of the gene product. See Table S9 for listing of networks.

We used the IPA^®^ tool to elucidate the interactions between the target genes of the four TFs to investigate how these genes may contribute to the biological mechanisms of AAA. The IPA’s Core Analysis generated 25 networks (Table S9), the top five of which were (1) “*cellular function and maintenance*, *hematological system development and function*, *hematopoiesis*” (score: 41); (2) “*respiratory system development and function*, *tissue morphology*, *cardiovascular system development and function*” (score: 41); (3) “*cell morphology*, *cell-to-cell signaling and interaction*, *developmental disorder*” (score: 39); (4) “*embryonic development*, *cellular function and maintenance*, *hematological system development and function*” (score: 36); and (5) “*cellular function and maintenance*, *cell death and survival*, *cardiovascular disease*”(score: 34). We then merged the networks into a single interaction figure indicating the genes with significantly elevated or decreased expression levels according to our prior microarray-based mRNA expression study (Figure 6) [7].

We carried out additional functional analyses with the IPA^®^ tool. The top canonical pathways were: (1) “p70S6K signaling” (*p* = 2.52 × 10^−06^); (2) “*CCR5 signaling in macrophages*” (*p* = 4.88 × 10^−06^); (3) “*protein kinase A signaling*” (*p* = 5.15 × 10^−06^); (4) “*hepatic fibrosis/hepatic stellate cell activation*” (*p* = 5.18 × 10^−06^); and (5) “*RhoA signaling*” (*p* = 1.37 × 10^−05^). The five most significant “molecular and cellular functions” were: (1) “*cellular development*” (378 molecules); (2) “*cellular growth and proliferation*” (406 molecules); (3) “*cell morphology*” (319 molecules); (4) “*cellular movement*”(266 molecules) and (5) “*cellular function and maintenance*” (380 molecules).

**Figure 7:**
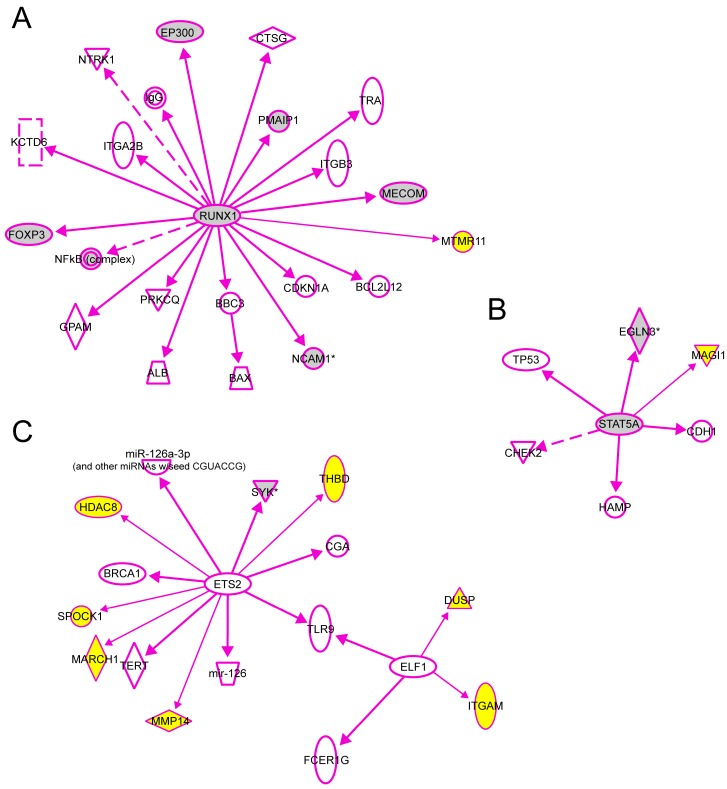
Experimentally validated direct interactions of transcription factors (**A**) RUNX1, (**B**) STAT5 and (**C**) ELF1, ETS2 with their target genes. Qiagen’s Ingenuity Pathway Analysis^®^ tool was used to demonstrate the downstream interactions of TFs to genes with experimental evidence that their transcription is regulated directly by one of the TFs studied here. In addition, the genes *MTMR11*, *DUSP10*, *ITGAM*, *MARCH1*, *HDAC8*, *MMP14*, *MAGI1*, *THBD* and *SPOCK1* identified as target genes for these TFs in human aorta in the current study using ChIP-chip and q-PCR were also included (yellow symbols). Eight of the previously known target genes (gray symbols) were also identified in the current ChIP-chip dataset. Two of the target genes shown in this figure (*SYK* and *PMAIP1*) had significantly different mRNA levels between AAA and control aorta [7].

We also used the IPA tool to demonstrate the downstream interactions of TFs to genes with experimental evidence that their transcription is regulated directly by one of the TFs studied here. There were a total of 20 genes regulated directly by RUNX1, 7 with ETS2, 5 with STAT5 and 2 with ELF1 (Figure 7). We then added to these interaction graphs shown in Figure 7 the genes *MTMR11*, *DUSP10*, *ITGAM*, *MARCH1*, *HDAC8*, *MMP14*, *MAGI1*, *THBD* and *SPOCK1* (shown with yellow background in Figure 7) identified as target genes for these TFs in the human aorta in the current study using ChIP-chip and q-PCR. Eight of the previously known target genes (gray symbols) were also identified in the current ChIP-chip dataset. Two of the target genes shown in Figure 7 (*SYK* and *PMAIP1*) had significantly different mRNA levels between AAA and control aorta [7].

RUNX1, ELF1, ETS2, and STAT5 are important regulators of immune cell proliferation, differentiation, and activation [14,34,35,36]. Further investigation on the pattern of RUNX1, ETS2, and STAT5 expression early in AAA pathogenesis may clarify these roles. RUNX1, STAT5, and ELF1 may play crucial roles in the immune response seen in AAA.

**Figure 8:**
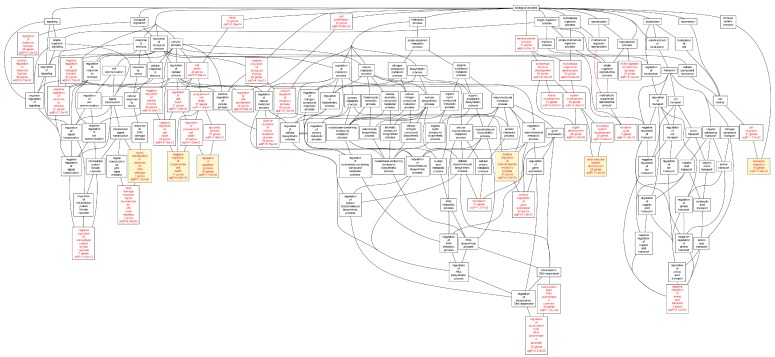
Biological categories of the target genes of transcription factors RUNX1, ELF1, ETS2 and STAT5 that were also differentially expressed in the AAA neck region. The gene list was obtained from a previously published study on RNA samples isolated from the neck regions of AAAs [40]. A DAG of the GO categories of the set of 282 genes was generated by the web application WebGestalt [32]. Categories shown in red were significant (adjusted *p* < 0.001). The categories on the 6th level of hierarchy are discussed in the text and are shown here with a yellow background. See Tables 12 and S11 for additional information.

Several intriguing observations of genes with associated specific chip enriched regions may have relevance to AAA pathogenesis. Two RUNX1 and one ETS2 chromatin enriched regions were associated with CD59, a suppressor of the complement response, in control samples. Only one RUNX1 chip-enriched region was present in the AAA (Table S1). Inhibition of CD59 attenuates aneurysm formation in the angiotensin mouse model of AAA [37]. DUSP10 up-regulated in AAA had both ETS2 and ELF1 binding sites (Table 4; Table S1). DUSP10 (Dual specificity protein phosphatase 10) regulates magnitude of p38 activity in response to oxidative stress in HEK 293-T cells [38]. ETS2 was enriched near the MMP14 locus (Table 4; Table S1). Previously ETS protein inhibitors were suggested to reduce MMP expression [39]. Further investigation may determine if this pattern represents a cell type specific transcription factor binding pattern or if it represents combinatorial control of gene expression. Other genes found in our ChIP-chip analysis are involved in immune activation and apoptosis; however a complete discussion on the relevance of these genes to AAA is outside the scope of our analysis (Figure 2 and Figure 8).

### 2.5. Analysis of mRNA Expression Data from the Neck Region of AAA

Biros *et al.* [40] studied gene expression in the aneurysmal neck and identified 1047 differentially expressed genes (≥2-fold difference and FDR < 0.05) in comparison with juxtarenal aortic tissue samples obtained from age-and sex-matched kidney donors. These authors hypothesized that by using the non-dilated neck region of AAA, they would be able to study biological processes involved in early stages of AAA development [40]. Overall, there were only 88 genes that were common in the differentially expressed gene lists of Lenk *et al.* [7] and Biros *et al.* [40]. We also analyzed the list of 1047 genes from the Biros *et al.* [40] study and found 446 genes with chers for TFs RUNX1, STAT5, ELF1 or ETS2 (Table S10). The most enriched GO categories included “*regulation of apoptosis*” (FDR = 1.50 × 10^−03^), “*positive regulation of macromolecule metabolic process*” (FDR = 2.10 × 10^−03^), “*cardiovascular system development*” (FDR = 3.50 × 10^−03^) and “*negative regulation of programmed cell death*” (FDR = 9.00 × 10^−03^) (Table 12, Figure 8 and Table S11). It is noteworthy that these functional categories were different than the ones described above with the aneurysmal sac samples. These results suggest that apoptosis is an earlier event than inflammation in the AAA development.

**Table 12:** Enriched GO biological process categories for the target genes differentially expressed in the AAA neck and found to have binding sites for transcription factors RUNX1, ELF1, ETS2 and STAT5 based on the current ChIP-chip analyses.

GO Biological Process Category	GO ID	Number of Genes			*p*	FDR
		Reference Set	AAA Neck Set	Expected		
Regulation of apoptotic process	0042981	1150	30	13	5.72 × 10^−06^	1.50 × 10^−03^
Positive regulation of macromolecule metabolic process	0010604	1842	40	20	1.10 × 10^−05^	2.10 × 10^−03^
Cardiovascular system development	0072358	754	22	8.2	2.25 × 10^−05^	3.50 × 10^−03^
Negative regulation of programmed cell death	0043069	580	17	6.3	2.00 × 10^−04^	9.00 × 10^−03^
Signal transduction in response to DNA damage	0042770	116	7	1.2	3.00 × 10^−04^	1.21 × 10^−02^
Leukocyte migration	0050900	248	10	2.7	4.00 × 10^−04^	1.43 × 10^−02^

The gene list was obtained from a previously published study on RNA samples isolated from the neck regions of AAAs [40]. From this list, genes enriched in the ChIP-chip experiments identified in the current study were identified. This gene set (see Table S9) was then compared to the reference gene set in the human genome annotated to at least one GO category. The analysis was carried out using a set of chers in the AAA sample pool. The analyses presented here were carried out using the combined set of all target genes for all four transcription factors. All of the categories listed here are from the 6th level of hierarchy. Size of the category (number of genes) and the number of genes overlapping with the list of genes are given. The analysis was carried out using the Webgestalt tool [31,32]. *p* values were calculated using a hypergeometric test and adjustment for multiple hypothesis testing (FDR) was performed using Benjamini-Hochberg correction [33]. See Figure 8 for a DAG image of the categories, and Table S11 for a list of the genes in the aorta set in each category.

### 2.6. Limitations of the Study

We realize that experimental cataloging of the target genes of each TF is only the first step in the process of understanding transcriptional regulation contributing to human diseases [10,41]. The binding of TFs to their target sequences can be influenced by many factors including the specificity of the binding site sequence, differences in the chromatin structure, and availability of co-factors, including co-activators and repressors increasing the complexity of the transcriptional control [42]. The chromatin structure can be influenced by post-translational histone modifications and DNA methylation [42]. According to previous studies, members of the ETS and STAT protein families often bind to relatively weak binding sites because they are part of protein complexes, which relaxes the requirement for a strong TFBS [13,43]. ELF1, although a member of the ETS TF family, is an exception in that it has stricter core binding motif requirements [13,44]. RUNX1 is known to form a complex with CBFB (core-binding factor, beta subunit), which does not, however, bind directly to the DNA and primarily stabilizes RUNX1 binding [45].

As pointed out by Todeschini *et al.* [46] in a recent review article, the “footprints of TF binding” (as they called them) need to be combined with dynamic information to distinguish specific functional binding of TFs with a direct consequence in gene expression from non-functional binding, either specific or non-specific, without influencing the transcriptional control. ChIP-chip results show where TFs bind, but provide no kinetic information on the binding. They are influenced by experimental details such as how long the chromatin was exposed to the fixative as well as bioinformatics tools and algorithms used to detect the peaks [46].

In the current study, we chose four TFs for the ChIP-chip based on our previous *in silico* analyses [8]. It is, however, likely that many additional TFs are important in the transcriptional regulation of human AAA. Some of these TFs are yet to be discovered as TFs [9]. In addition, performing ChIP-seq, in which the ChIP is followed by whole genome sequencing would allow cataloging of all TFBSs and not limiting to those present on the microarray used for hybridization [47].

The results discussed here are based on experiments with human aortic tissue samples from patients affected with AAA in the infrarenal aorta and age- and sex-matched controls without AAA. We considered it important to use the aortic tissue, since large differences have been reported in the number of putative binding sites for different TFs and cell types [42]. We acknowledge the limitations of the study due to limited availability of human aortic tissue from AAA patients and that samples can only be obtained at the end-stage of the disease when the AAA is large enough to warrant a surgical repair. At this stage it is difficult to collect samples from the different layers (intima, media and adventitia) of the aortic wall in large enough quantities for the type of experiments described here, due to extensive degradation and remodeling of the aorta. Nevertheless, the results provide intriguing preliminary results on the transcriptional control of gene expression in the human AAA. By combining the ChIP-chip results with the available mRNA expression profiles for AAA and control aorta we were able to generate more meaningful results related to the transcriptional control of gene expression in human AAA [48].

## 3. Experimental Section

### 3.1. Human Aortic Samples

Full thickness aortic wall tissue specimens were collected from patients undergoing AAA repair operations (*n* = 6) at the Geisinger Medical Center, Danville, PA, USA (Table 2). Non-aneurysmal aortic samples (*n* = 5) of the infrarenal aorta were collected at autopsies at Wayne State University School of Medicine, Detroit, MI, USA (Table 2). Tissue samples for ChIP were snap-frozen in liquid nitrogen and stored at −80 °C. The same autopsy samples have been used in our previous studies and have shown comparable performance in mRNA and protein analyses to samples taken from AAA operations [14,49,50,51,52]. The investigation conformed to the principals outlined in the Declaration of Helsinki. AAA patients gave written informed consent for the use of their aortic tissue samples for research. The collection of the human tissues and their use for research was approved by the Institutional Review Boards of Geisinger Health System, Danville, PA, USA, and the Wayne State University School of Medicine, Detroit, MI, USA.

### 3.2. Cell Culture Experiments

Human aortic smooth muscle cells (HASMC; catalog number 6110, ScienCell Research Laboratories, Carlsbad, CA, USA), human aortic endothelial cells (HAEC; catalog number 6100, ScienCell), and monocyte/macrophage cells (THP-1 cell line; catalog #TIB-202, American Type Culture Collection, Manassas, VA, USA) [53] were cultured in appropriate medium according to suppliers’ recommendations [14,50]. HASMCs and HAECs were stimulated for 18 h using 50 ng/mL IFN-γ; (PeproTech Inc., Rocky Hill, NJ, USA) as reported previously [21]. THP-1 cells [53] were stimulated for 18 h using 50% serum, 100 ng/mL lipopolysaccharide LPS (serotype 055: B5, Sigma-Aldrich, St. Louis, MO, USA), and 20 ng/mL IFN-γ (PeproTech) [22]. This stimulation protocol has been used previously [14,22] and was considered relevant to AAA, since mice lacking IFN-γ are resistant to AAA formation in the CaCl_2_ model [54] and IFN-γ-producing T-cells are present in the blood and aortic wall of most AAA patients [55].

### 3.3. Chromatin Immunoprecipitation Followed by Microarray Hybridization (ChIP-Chip)

ChIP was performed using EZ-ChIP Kit (Millipore, Billerica, MA, USA). In cultured TPH-1 cells DNA-protein complexes were cross-linked and then sheared to 200–1000 bp using a micro-tip equipped Sonicator 3000 (Misonix Inc., Farmingdale, NY, USA).

For aortic samples (Table 2) two pools, “AAA Pool” and “Control Pool”, were formed. A total of 65 g of frozen tissue from control (*n* = 5) or AAA (*n* = 6) samples (approximate equal weight of each) was combined and cut into 1–3 mm pieces. One mL of phosphate buffered saline (PBS) with CompleteMini Protease inhibitors (Roche, Mannheim, Germany) was added for each 100 mg of tissue. Crosslinking was performed by adding 37% formaldehyde to a final concentration of 1.5%. Samples were incubated at room temperature with rotation for 15 min. Cross-linking was stopped by adding glycine to a final concentration of 0.125 M. Samples were centrifuged and washed with ice-cold PBS and suspended in 100 μL of PBS with CompleteMini Protease inhibitors for each 100 mg of tissue. The tissue suspension was placed into a 50 μm disposable polyethylene chamber containing an immobile stainless steel screen with approximately 100 hexagonal holes, each with 6 microblades designed for efficient cutting of hard tissues (Medicon from BD Biosciences, San Jose, CA, USA). The Medicon was then inserted into an automated, mechanical disaggregation machine (Medimachine from BD Biosciences) for two min and finally filtered using a 50 μm disposable filter devise (Cup Filcon from BD Biosciences). The resulting cell suspension was centrifuged for 10 min, supernatant was removed and cell pellet was suspended in 500 μL lysis buffer (50 mM Tris-HCl, pH 8.1, 10 mM EDTA, 1% SDS). Sheared crosslinked chromatin was generated by sonicating cell lysates using a micro-tip equipped Sonicator 3000 (Misonix Inc.). The sonicated sample was centrifuged for 10 min at 10,000× *g*, and supernatant stored at −80 °C in 100 μL aliquots.

Immunoprecipitation of crosslinked chromatin and purification of DNA was performed using the EZ-ChIP Kit (Millipore). Briefly, specific antibodies or control antibodies (anti-RNA polymerase or normal mouse IgG) were added to diluted agarose G precleared aliquots and incubated overnight. Agarose G was added and the antibody-antigen-DNA complex was collected by centrifugation. Pellets were washed with a series of wash buffers and protein-DNA complexes eluted with elution buffer. Crosslinks of the eluted protein-DNA were reversed and the sample was first treated with RNase and then proteinase K. DNA was purified using spin columns.

The following experimental antibodies were used for immunoprecipitations: (1) purified mouse STAT5 antibody (catalog #610191; BD Transduction Laboratories, San Jose, CA, USA) [14]; (2) rabbit polyclonal ELF1 antibody (C-20X; sc-631, Santa Cruz Biotechnology, Inc., Santa Cruz, CA, USA) [8,23]; (3) rabbit polyclonal ETS2 antibody (C-20X; sc-351; Santa Cruz Biotechnology) [24,25,26]; and (4) purified rabbit polyclonal AML1 (also known as RUNX1) antibody (catalog #PC284; Calbiochem, Darmstadt, Germany) [27]. The literature citations indicate other studies that have used exactly the same antibodies.

Purified DNA was first amplified using GenomePlex^®^ Complete Whole Genome Amplification (WGA) Kit (Sigma-Aldrich, St. Louis, MO, USA). GenomePlex^®^ WGA Reamplification Kit (Sigma-Aldrich) was used in instances when a second amplification was warranted.

Amplified input and chromatin-immunoprecipitated DNA samples were used in hybridizations with promoter arrays (HG18 Deluxe Promoter HX1 arrays, Roche NimbleGen, Inc., Indianapolis, IN, USA). These arrays contain 2.1 million probes of 50–75 mer in size and tile the promoter regions of all known genes covering a genomic region of approximately −7 to +3 kbp for each gene. Labeling, hybridization, washing, and scanning were performed by NimbleGen. Signal intensity data were extracted from scanned images using NimbleScan software. For each probe on the array, log_2_ ratios of the Cy5-labeled IP sample *vs.* the Cy3-labeled input DNA sample were calculated.

### 3.4. Analysis of Chip Data from Chromatin Immunoprecipitation

The raw ChIP-chip data (*.pair*) and reporter probe IDs were loaded into statistical program R using the Ringo package [56]. The *pos* file provided by NimbleGen contained genomic mapping of the reporters to the hg18 version of the human genome. The *ndf* file contained the reporter sequences which were used to realign the reporter probe target locations to the hg19 version of the human genome using the alignment tool Exonerate [57,58]. Several methods part of the Ringo package [56] were used to assess the quality of the microarray data. The data passed these quality control steps.

The NimbleGen reporter annotation field contained 2,125,410 reporter targets for the hg18 version of the human genome. The reporters mapped to 2,190,504 (65,094 or 3.1% more) genomic locations when remapped to the hg19 version of the genome. We removed all non-specific reporters (101,622 non-unique probes; 4.6%) to control error introduced by them, leaving 2,088,882 unique reporters. A total of 31,522 (1.5%) unique probes were removed from the original set of 2,125,410 probes.

We normalized the data according to the NimbleGen recommendation by using the Tukey-biweight scale normalization, which gives a robust average that reduces the effect of outliers. We smoothed the probe intensities as suggested by Ringo to account for parameters that influence genomic DNA hybridization to the microarray such as probe GC content, melting temperature, and secondary structure that can cause variation in individual probe-genomic fragment hybridization [56]. The reporter intensities were smoothed by calculating a running median using a 900-bp sliding window and a minimum of seven probes [59].

Chers were identified as regions containing at least seven probes with a mean enrichment value that exceeded the threshold (*y*_0_) and that were separated by at least 450 bp from another cher. The threshold was defined as the top 99th quantile (top 1%) of potential chers to limit the number of false positives included in the analysis.

### 3.5. Transcription Factor Binding Site (TFBS) Analysis

We analyzed the sequences of the chers to identify potential TFBS and support the evidence of TF binding from the ChIP-chip analysis. The program Ringo was used to generate a score which is the sum of the log-fold changes minus the threshold. We used the R package Biostrings to query the genomic dataset (hg19) for the sequences of chers and export them in a *fasta* file. The Match™ tool [60] of Transfac^®^ (Wolfenbuettel, Germany) was used to compare this dataset with a truncated version of the minFP_best (cut-off to minimize false positive matches) and minSum_best (cut-off to minimize the sum of both false positive and false negative matches) profiles containing only the TFs ELF1, ETS2, RUNX1 and STAT5.

### 3.6. Calculating Distance between Chip-Enriched Region (cher) and TFBS

After identifying the chers containing motifs for the TFBSs, we determined the distance from the predicted site to the TSS. We used the most proximal TSS for each gene. The distance between the TSS and TFBS was calculated as “(TSS − Cher_start_ + TFBS) × Strand”.

### 3.7. Gene Expression Analysis

Previously our laboratory generated genome-wide microarray-based mRNA expression profiles for both aneurysmal and non-aneurysmal human infrarenal abdominal aorta [7]. The microarray data can be obtained at the Gene Expression Omnibus (GEO) database (Series# GSE7084) [28,29]. We used this data set here for the target gene analysis (see below).

Another expressed gene list was obtained from a previously published study on RNA samples isolated from the neck regions of AAAs [40]. The authors generated this gene list by comparing the mRNA expression of aortic neck samples from AAA patients (*n* = 14) and control aortic specimens (*n* = 8) obtained from organ donors. The microarray platform was HumanHT-12 v4 Expression BeadChip (Illumina). Genes with 2-fold difference in expression between AAA and control groups and FDR < 0.05 were considered differentially expressed. A total of 1047 differentially expressed genes were identified [40].

### 3.8. Functional Classification and Network Analysis of Target Genes

Functional classification of the target genes was carried out with GO analysis using WebGestalt to create a hierarchy of the GO annotations of the predicted targets [31,32]. For this procedure, a list of the Entrez IDs for target genes that contained chers and were known to be differentially expressed based on our previous study [7] or that of Biros *et al.* [40] was uploaded to the web application WebGestalt Gene Set Analysis Toolkit Version 2 [32]. The analyses were carried out for each TF separately. Combined analyses with all differentially expressed target genes in either the AAA or control aorta were also performed, as well as with target genes of different combinations of the TFs. Directed acyclic graphs (DAGs) were generated representing a hierarchical categorization of the significant GO annotations.

KEGG pathway [61] analysis was carried out to identify enriched pathways for the combined set of target genes in AAA and control as well as for the AAA and control target genes separately using the R package KEGG.db [62].

Potential target gene interactions were analyzed via networks generated using Ingenuity Pathway Analysis^®^ (IPA) tool version 9.0 (Qiagen’s Ingenuity Systems, Redwood City, CA, USA).

### 3.9. Quantitative PCR (qPCR) Validation of Selected TFBSs

Predesigned ChIP-qPCR assays and RT^2^ SYBR Green/Rox qPCR Master Mix available from SABiosciences (Qiagen, Frederick, MD, USA) were used to validate selected ChIP-chip results. The assay IDs and their relationship with the chers identified in ChIP-chip are shown in Table 4. Positive [GPH10001C(+)01A] and negative (IGX1A) control assays available from SABiosciences were also included. All assays were run according to manufacturer’s recommendations.

## 4. Conclusions

The ChIP-chip-based transcriptional profiling of human aortic tissues demonstrated large differences between the aneurysmal (AAA) and non-aneurysmal infrarenal human aorta. Most likely the TFs are not acting alone, but changes in DNA architecture including histone modifications, invoke different transcriptional responses [63,64]. In addition, TFs often bind in a combinatorial fashion, as revealed by the ENCODE project [63], and it will, therefore, be important to consider co-operative binding of TFs in future experiments. Furthermore, it is plausible to speculate that TFBSs harboring sequence variations in humans could provide mechanisms in disease pathogenesis as has been shown for the sequence variant located on chromosome 9p21 and associated with multiple forms of vascular diseases [64].

## Supplementary Materials

Supplementary materials can be found at http://www.mdpi.com/1422-0067/16/05/11229/s1.
